# High Fat Programming and Cardiovascular Disease

**DOI:** 10.3390/medicina54050086

**Published:** 2018-11-13

**Authors:** Marlon E. Cerf

**Affiliations:** 1 Biomedical Research and Innovation Platform, South African Medical Research Council, Tygerberg 7505, South Africa; marlon.cerf@mrc.ac.za; Tel.: +27-21-938-0818; 2 Division of Medical Physiology, Department of Biomedical Sciences, Faculty of Medicine and Health Sciences, University of Stellenbosch, Tygerberg 7505, South Africa

**Keywords:** fatty acids, maternal obesity, miRNAs, offspring cardiac outcomes

## Abstract

Programming is triggered through events during critical developmental phases that alter offspring health outcomes. High fat programming is defined as the maintenance on a high fat diet during fetal and/or early postnatal life that induces metabolic and physiological alterations that compromise health. The maternal nutritional status, including the dietary fatty acid composition, during gestation and/or lactation, are key determinants of fetal and postnatal development. A maternal high fat diet and obesity during gestation compromises the maternal metabolic state and, through high fat programming, presents an unfavorable intrauterine milieu for fetal growth and development thereby conferring adverse cardiac outcomes to offspring. Stressors on the heart, such as a maternal high fat diet and obesity, alter the expression of cardiac-specific factors that alter cardiac structure and function. The proper nutritional balance, including the fatty acid balance, particularly during developmental windows, are critical for maintaining cardiac structure, preserving cardiac function and enhancing the cardiac response to metabolic challenges.

## 1. Introduction

### 1.1. The Programming Concept and Cardiovascular Disease

Programming is triggered through events during critical developmental phases that alter offspring health outcomes. Fetal programming refers to exposure to a stimulus or insult during fetal (in utero) life. Lactational programming refers to exposure to a stimulus or insult during early postnatal life i.e., from birth up to weaning. Developmental programming spans both fetal and lactational programming i.e., from fetal life up to weaning. Most animal studies focus on either fetal or lactational programming, the latter can be extended to include childhood. The preconception phase and paternal contribution to offspring health outcomes are also recognized as forms of programming.

Table 1 summarizes the consequences of fetal programming. There are diverse programming effects that induce specific metabolic derangements that have been demonstrated across species. Some programming effects include altered expression profiles of growth, proliferation and circadian rhythm factors, altered antioxidant enzyme actions, and the induction of increased atherogenesis, blood pressure, vascular activity and heart rate.

### 1.2. Overview of High Fat Programming

Nutrition is a programming stimulus or insult that can typically be low/high protein, carbohydrate or fat diets, or combinations, administered during specific life stages (i.e., developmental windows) [24]. Maternal nutrition and dietary fatty acid (FA) composition during gestation and/or lactation are key determinants for normal fetal and postnatal development and contribute to fetal programming effects that typically influence the offspring’s susceptibility to metabolic diseases over the life course [25]. High fat programming is defined as the maintenance on a high fat diet (HFD) during fetal and/or early postnatal life that induces metabolic and physiological alterations that compromise health. Specifically, high fat programming refers to the exposure of offspring to a high saturated fat diet during fetal and/or lactational life via maternal nutrition [24]. The HFD is typically high in saturated fat content, usually ≥40% saturated fat (as energy), that is often derived from animal fat sources. However, some programming effects can be observed from ≥30% fat (as energy) diet depending on the dietary constituents. The proportions of the other macronutrients viz. carbohydrates and protein are adjusted due to the increase in fat content in the diets. However, protein content is usually maintained at high enough levels i.e., ≥15% protein as energy to avoid the adverse programming effects of protein deficiency. Glucolipotoxicity refers to the simultaneous elevation of glucose and lipids that results in intracellular accumulation of lipids and lipid metabolites, with adverse effects on pancreatic [26] and cardiac structure, function and survival. The exposure of the fetus to hyperglycemia and hyperlipidemia implicates glucolipotoxicity [27] in the onset of metabolic diseases such as cardiovascular disease (CVD). 

### 1.3. High Fat Programming of Cardiovascular Disease

Cardiac insulin signaling can be impaired due to a shift in FA metabolism resulting in insulin resistance. A maternal HFD diminishes cardiac function in offspring exposed to diabetic pregnancy through metabolic abnormalities, oxidative stress and mitochondrial dysfunction [28]. Further, a maternal HFD compromises organ development and renders the offspring prone to metabolic diseases later in life including CVD [29]. Maintenance on an HFD during gestation altered the placenta resulting in fetuses that were either smaller or larger [29]. In rodent models, it was revealed that maternal obesity adversely impacted the offspring, evident by hypertension, adiposity, hyperphagia, dyslipidemia, insulin resistance and hepatic steatosis [30,31,32]. Primates closely mimic human obesity, diabetes and CVD in pathology, altered glycemia and complications [16]. In primates, HFD maintenance during pregnancy led to broad developmental health issues in the offspring [3,33,34,35,36,37]. The high fat programming effects in primate offspring were restricted fetal growth, placental insufficiency with reduced placental blood flow volume, increased cytokine release, dyslipidemia and increased hepatic fat deposition [3,33,34,35,36,37]. Postnatally, the offspring maintained on an HFD displayed catch-up growth, increased fat mass and persistent fatty liver reflecting an increased risk for CVD. In primates, maintenance on an HFD (through maternal feeding and lactation) impaired offspring vascular function evident by diminished endothelium-dependent vasodilatation, thickened intima walls, and the onset of inflammation and prothrombosis which predisposes them to an increased risk of early-onset atherogenesis [16]. Importantly, the vascular function impairments observed in HFD offspring presented prior to development of obesity [16].

Prenatal insults that adversely affect fetal growth increase the incidence of hypertension in adulthood [38,39,40]. This demonstrates the early initiation of CVD and how it manifests later in life. The unfavorable intrauterine milieu can induce cardiac derangements that prompt the onset of CVD in adulthood. Maternal and early postnatal nutrition can alter the trajectories of metabolic and neural pathways involved in energy homeostasis, linked to the programming of obesity, thereby leading to disease in adulthood [41].

### 1.4. Maternal Obesity

Maternal obesity adversely impacts the heart and is associated with CVD in both human and animal offspring [42]. Offspring of overweight and obese mothers are more susceptible to cardiovascular anomalies [43], with obesity during pregnancy associated with a higher risk of CVD in adult human offspring [44]. Further, the programming of hypertension may initiate during intrauterine life; however, it may also emerge over the offspring’s life course [38]. Low birth weights followed by altered subsequent growth trajectories (such as catch up growth) contributes to cardiovascular morbidities [38]. Atherosclerosis has a lengthy asymptomatic phase [45] as pathological manifestations emerge in the arteries of children and young adults, decades prior to overt clinical atherosclerosis [45]. Early life (in utero, infancy and childhood) nutritional factors determine the progression of atherosclerosis [45]. Maternal hypercholesterolemia, obesity and diabetes particularly increase CVD risk [39,46] over the life course of offspring. There are also global changes in fetal lipid and mitochondrial metabolic pathways that predict later onset metabolic disease, obesity [47] and CVD. The maternal programming influences, viz. maternal HFD consumption and obesity, are discussed in the context of offspring cardiovascular outcomes.

## 2. High Fat Programming: Sex-Specificity, Altered Cardiac Gene and microRNA Expression, and Modified Cardiac Structure and Physiology

### 2.1. Sex-Specific Influences

Differential sex mechanisms of fetal cardiac programming are caused by an adverse intrauterine milieu [48] such as high fat programming. In offspring, sex-specific metabolic derangements are triggered by high fat programming [49], and although conflicting, males are more susceptible to the programming of CVD [50]. Maternal hyperglycemia in pregnancy is independently associated with the offspring’s risk for glucose intolerance, obesity and an increase in blood pressure in seven-year-old children, with only girls being obese [51]. In female sheep, after prenatal bisphenol A (BPA, commonly found in polycarbonate plastic and epoxy resins) exposure, postnatal overfeeding/adiposity, and the combination, led to myocardial transcriptional changes, and despite different gene profiles, still influenced similar signaling pathways [21]. Genes altered by the programming insult were implicated in obesity, hypertension or heart disease [21]. In rats, a maternal HFD causes cardiac hypertrophy; increases cardiac susceptibility to ischemic-reperfusion injury only in adult male offspring, and differentially regulates cardiac angiotensin II (AngII) receptor type 1 (AGTR1) and type 2 (AGTR2) expression [48]. Multiple mechanisms are involved in the sex-specific effects of maternal HFD [48].

### 2.2. Altered Cardiac Gene and miRNA Expression

AngII, a hormone that regulates blood pressure, and its receptors, viz. AGTR1 and AGTR2, play key roles in the regulation of cardiovascular homeostasis and are implicated in the fetal programming of cardio-cerebrovascular diseases [48,52,53,54,55]. Further, upregulation of AGTR2 played a causal role in maternal HFD-induced higher cardiac susceptibility to ischemia-reperfusion injury in male offspring; and decreased glucocorticoid receptors (GR) binding to glucocorticoid response elements (GREs) at the Agtr2 promoter through HFD-mediated Agtr2 gene upregulation [48]. Therefore, a maternal HFD influences the expression of key blood pressure regulatory factors that alter cardiac structure and susceptibility to ischemia-reperfusion injury.

There is an intricate cardiac miRNA network, modulated by HFD administration, and implicated in the pathogenesis of CVD. Poor cardiac outcomes are linked to miRNAs modulated after HFD exposure in mice [56]. In young adult mouse offspring (12 weeks of age), exposure to maternal obesity altered some cardiac-relevant microRNA (miRNA, small, non-coding endogenous RNAs of 21–25 nucleotides) expression, overlaid on a cardiac miRNAome that was markedly changed after 9 weeks of exposure to a HFD (the HFD mimicked a Western fast-food diet) [56]. After 9 weeks of the HFD administration, 33 cardiac miRNA expression profiles were altered [56]. miR-30 family members were prominent as their dysregulation is associated with cardiac abnormalities and ischemia-reperfusion injury [57], cardiomyocyte endoplasmic reticulum (ER) stress [58] and pathological hypertrophy [59,60]. Diet directly impacts cardiac miR-126 (a negative regulator of insulin signaling) expression [56] evident by its 2.5-fold upregulation induced by diet-induced maternal obesity [61]. miR-499 overexpression (>2 fold after HFD exposure) augmented heart mass and hypertrophy and exacerbated contractile dysfunction [62]. Both miR-24 and miR199a were overexpressed in failing human hearts, murine cardiac hypertrophy, and induced hypertrophy in vitro [63]. About two-thirds of miRNAs were diet-upregulated >2 fold in failing hearts [64], therefore the miRNAs altered by poor nutrition, such as exposure to a HFD in utero, are likely implicated in poor cardiac health outcomes [56]. miR-208a-3p was upregulated >3 fold by the HFD [56]. The key cardiac-specific miR-208a is required for electrical conduction but its transgenic overexpression induces hypertrophy [63,65]. Cardiac-specific miR-208a knockdown improves whole-body energy homeostasis, yielding leaner mice resistant to diet-induced obesity [66]. The alterations in cardiac miRNA expression profiles by a HFD demonstrates the importance of proper nutrition particularly during critical developmental periods. Maternal nutrition during gestation and lactation sustains and shapes offspring growth and therefore the correct healthy balance of nutrients is necessary for positive offspring development, growth and health outcomes.

The intrauterine milieu can considerably alter fetal genome expression thereby stimulating or inhibiting fetal growth and adiposity [67]. Downregulation in the miR-17-92 cluster resulted in septal defects in mice [68] whereas miR-181a plays a role in cardiac neural crest migration [69]. The dysregulation of developmentally key and relevant miRNAs may explain why the offspring of obese women are more susceptible to several congenital heart defects [70,71]. Differential miRNA expression, through developmental programming such as maternal high fat feeding and obesity, therefore influences cardiac development and health outcomes in offspring.

Some miRNA expression altered by a maternal HFD reflected expression profiles in adult cardiac diseases, such cardiac hypertrophy (miR-21, miR-143 and miR-499) [72], heart failure (miR-21 and miR-223) [73] and myocardial infarction (miR-30c, miR-139 and miR-451) [72]. Overexpressed miRNAs were implicated in increasing fibrosis (miR-21, miR-499, miRs-30 family and miRs-133 family) [74], intracellular trafficking and cell adhesion (miR-30 family) [59]. Altered cardiac miRNA expression likely diminishes cardiac function in offspring maintained on a HFD [67]. A maternal HFD, administered preconception and during gestation, programmed fetal cardiac fibrosis concomitant with differential cardiac miRNA expression likely implicated in the programming of impaired cardiac development [67]. Altered miRNA expression, through HFD modulation, therefore alters cardiac structure with implications on cardiac function and health.

### 2.3. Modified Cardiac Structure and Physiology

A maternal HFD had no effect on body weight but increased the heart weight and heart weight-to-body weight in the adult male offspring, which was supported by the echocardiographic (ECG) analysis showing increased wall thickness and decreased left ventricle internal diameters [48]. Thus, heart weight or its ratio to body weight (independent of body weight), is important as body weight solely may not alter despite structural and potentially functional cardiac alterations. Further, a maternal HFD caused cardiomyocyte hypertrophy in adult male offspring rats [48] consistent with another study reporting that maternal overnutrition during gestation and lactation in mice induced cardiac hypertrophy in male offspring [75]. Alterations in the myocardium have also been reported in a primate model of fetal programming [16]. In rodents, early life overnutrition programmed cardiac hypertrophy and decreased myocardial vessel density during cardiac development [76]. In addition, ECG analysis showed that a maternal HFD maintained normal systolic and diastolic function, indicating that the concentric geometric remodeling with a reduction in left ventricle chamber size relative to wall thickness is an adaptation to preserve left ventricle pump function [48]. Thus, after maintenance on a maternal HFD, the heart adapts by remodeling as a compensatory mechanism to maintain function. However, this may only be temporary as cardiac dysfunction may ensue with a persistent HFD or from the later effects of programming that present over the life course i.e., adulthood and ageing.

Clinical and experimental studies observed that the perinatal period and early development milieu regulate metabolic predispositions in adulthood [77] and ageing. Several epidemiological studies reported that early nutrition may permanently influence body weight trajectories and cardiometabolic risks viz. hypertension, dyslipidemia and insulin resistance, that render individuals susceptible to develop CVD [78,79,80,81,82]. Genomic plasticity during neonatal life is necessary for orientating adult phenotypic traits, since epigenetic modifications may alter gene expression and prompt the pathogenesis of chronic diseases [77]. In rodents, early overnutrition by decreasing litter size [83,84] limits competition for milk during lactation thereby resulting in lactational overnutrition [85] which reflects lactational programming. A reduction in rodent litter size led to a ~30% increase in body weight at weaning that was maintained as the rodents matured, albeit to a lesser extent [86,87,88]. Several metabolic syndrome traits such as overweight, insulin resistance and hypertension were reported in postnatally overfed rats [83,89]. In adult postnatally overfed rats, impaired cardiac insulin signaling was demonstrated [90,91] and lactational overnutrition programmed cardiac gene expression, that may permanently alter cardiac structure and metabolism, impair cardiac contraction and predispose offspring to myocardial ischemia-reperfusion injury [87,92,93]. Therefore, overnutrition, that often prompts overweight and obesity, modulates the expression of key cardiac factors, which alters cardiac structure and function. Neonatal cardiomyocytes exposed to a HFD had lipid droplet accumulation and mitochondrial hyperplasia with increased oxidative stress (lipid peroxidation) [28]. Neonatal cardiomyocytes exposed to both a HFD and diabetes presented impaired metabolic fuel flexibility, mitochondrial dysfunction, and diastolic and systolic dysfunction [28]. The maternal metabolic milieu therefore shapes and predicts offspring cardiovascular outcomes.

## 3. Maternal Obesity Triggers Cardiovascular Disease

Obesity encompasses the dysregulation of several molecular pathways and organ systems, including adipose tissue, pancreas, liver, the central nervous system, gastrointestinal tract and microbiome [94], and the cardiovascular system. Maternal hyperglycemia increased offspring susceptibility to obesity, hypertension and glucose intolerance during early childhood; this was independent of maternal obesity, macrosomia and childhood obesity [51]. Although irregular glucose tolerance levels in children may be low, the cardiometabolic risk perpetually increases over the life course [51]. A high omega-6/omega-3 ratio is prothrombotic and proinflammatory which fuels the high prevalence of atherosclerosis, diabetes and obesity [95,96,97,98,99,100]. HFDs rich in omega-6 FAs increase susceptibility to obesity, leptin resistance and diabetes in humans and rodents [101,102]. In humans and animals, a high omega-6 FA intake and a high omega-6/omega-3 ratio stimulate weight gain, whereas a high omega-3 FA intake limits weight gain [94]. The increasing incidence in obesity is most pronounced during the reproductive ages [103] and, thus, more children are conceived from overweight/obese parents [56] which contributes to the global increased incidence and prevalence of obesity. However, early exposure to omega-3 FAs appears favorable for lipid homeostasis and catalase protein production in offspring during childhood [104]. The maternal intake of omega-3 FAs in normocaloric and normolipidic diets during gestation and lactation decreased serum concentrations of triacylglycerol (TAG) and total cholesterol [104]. Therefore, the quality of FAs influences offspring lipidemia with consequences for cardiometabolic health. 

Both epidemiological and experimental evidence demonstrate that overnutrition during intrauterine life, often accompanied by obesity, can program susceptibility to metabolic dysfunction in adulthood [105] and that an adverse intrauterine environment increases the risk for CVD in adulthood [106,107]. These adverse metabolic effects are genome-independent suggesting that the intrauterine milieu exerts its influences on offspring susceptibility to metabolic disease [56]. Maternal overweight and obesity are also considered as important risk factors for adult diseases in offspring, including metabolic disorders, type 2 diabetes, hypertension and CVD [108,109,110,111]. Maternal obesity is associated with various adverse outcomes for the mother, such as preeclampsia and gestational diabetes [48]. Maternal obesity and HFD consumption during gestation increase the risk of offspring obesity through complex mechanisms, which involve metabolic dysregulation and alteration of food intake behavior [112]. Exposure to maternal adiposity during gestation is linked to offspring with heavier birth weights and greater adiposity through childhood and over their life course [113]. Developmental overnutrition enables excessive transplacental passage of nutrients that results in larger babies with greater fat mass [114]. Metabolic dysregulation predisposes to CVD, and in humans, offspring from obese mothers are predisposed to CVD [44,115]. Animal studies have identified the risk of hypertension [116,117], vascular dysfunction [118], cardiac hypertrophy and contractile dysfunction [75,119] to all be shaped by maternal obesity during gestation [56]. Further, maternal obesity during gestation is associated with the adverse long-term offspring cardiovascular health outcomes, that may be independent of the genome and adult lifestyle choices [105]. High omega-6 FA intake during the perinatal period contributes to higher offspring adiposity [94]. Maternal macronutrient (HFD) and micronutrient (FA) profiles shape offspring cardiovascular outcomes. Maintaining the optimal nutrition to support the pregnant and lactating mothers’ dynamic metabolism is critical to ensure proper development, growth and maturation of their offspring.

## 4. Maternal High Fat Feeding and Obesity Influences on Offspring Cardiac Outcomes

**Figure 1:**
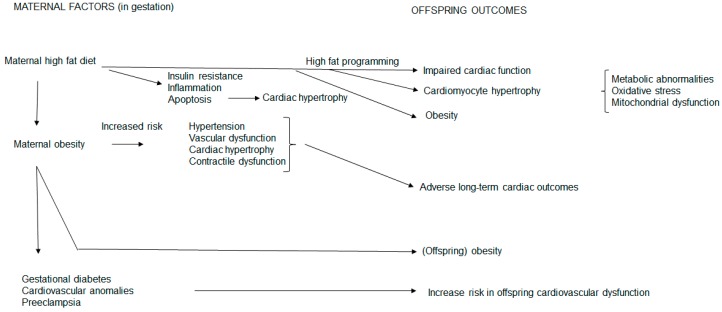
Maternal high fat feeding and obesity influences on offspring cardiac outcomes. A maternal high fat diet and obesity compromises the maternal phenotype and exposes the fetal offspring to an unfavorable intrauterine milieu. This prompts structural and functional cardiac alterations in offspring through conferrance of undesirable metabolic sequalae thereby increasing their risk for cardiovascular disease.

Figure 1 summarizes the influences of maternal high fat feeding on their offspring’s cardiac outcomes. Maternal factors, such as malnutrition during gestation, impair myocardial cardioprotection, resulting in cardiac vulnerability. Glucolipotoxicity, with excess circulating glucose and saturated FAs, induces adverse cardiac outcomes [120], therefore maintaining an optimal intrauterine milieu is important for the developing heart. High fat programming often presents a glucolipotoxic milieu, usually co-existing with maternal obesity, which translates into undesirable consequences for offspring health outcomes. Maternal hyperglycemia during gestation also independently contributes to the offspring’s risk of hypertension, glucose intolerance and obesity [51]. Thus, the heart is susceptible to structural and functional changes.

In the mother, a HFD during gestation induces insulin resistance, inflammation, apoptosis and cardiac hypertrophy. These unfavorable maternal metabolic derangements, therefore, present an undesirable intrauterine milieu for fetal development and growth. Hence a maternal HFD confers undesirable metabolic sequelae to offspring which influences their cardiac outcomes. This occurs through high fat programming and reflects maternal metabolic conferrance to the fetal offspring. In these offspring, the mother confers impaired cardiomyocyte hypertrophy and cardiac function concomitant with obesity. In addition, metabolic abnormalities, oxidative stress and mitochondrial dysfunction [28] also contribute to offspring cardiac hypertrophy, dysfunction and obesity. 

As a maternal HFD and maternal obesity are usually entwined, in the mother, maternal obesity increases the risk of gestational diabetes, increases cardiovascular anomalies and increases the risk of preeclampsia reflecting metabolic maternal derangements. Maternal obesity also increases the inherent risk of hypertension, vascular dysfunction, cardiac hypertrophy and contractile dysfunction that confers long-term adverse cardiac outcomes to offspring concomitant with obesity. 

Thus, the compromised maternal metabolic state during gestation, through maternal HFD consumption and obesity, programs adverse cardiovascular outcomes in offspring. At birth, offspring may be equipped with varying abilities to compensate in response to cardiac demand. However, some offspring may be more susceptible to develop CVD. Hence, prenatal, and later postnatal, nutrition are also critical periods for shaping offspring health outcomes.

## 5. Conclusions

Nutrition in pregnancy requires a careful balance of both the quality and quantity of fat intake to optimize fetal growth and development, also reducing maternal morbidity [121]. The maternal nutritional status and dietary FA composition during gestation and/or lactation shape offspring development with high fat programming conferring cardiovascular risk to offspring that may present at any time over the life course. A well balanced maternal diet during gestation and lactation, with a favorable FA balance, is therefore critical for good offspring cardiovascular health.

## Figures and Tables

**Table 1:** Programming of metabolic disease.

Programming Alterations	Species	References
Modified mRNA and protein expression of growth, proliferation and circadian rhythm factors	Mice, primates	[1,2,3]
Modified antioxidant enzyme actions	Mice	[2]
Modified inflammation due to altered mediators and regulators	Mice, rabbits	[2,4,5]
Modified metabolic glucose and insulin action	Mice, rats, humans	[2,6,7,8,9,10,11,12,13]
Modified cholesterol synthesis	Mice	[14]
Increased atherogenesis	Mice, rabbits, primates, humans	[4,15,16]
Elevated blood pressure and vascular reactivity	Mice, rats, sheep	[17,18,19,20,21]
Elevated heart rate	Mice, sheep	[22,23]

Specific programming effects on metabolic derangements are reported in mice, rats, rabbits, sheep, primates and humans.
